# Normal values for high-resolution anorectal manometry in healthy young adults: evidence from Vietnam

**DOI:** 10.1186/s12876-021-01865-8

**Published:** 2021-07-15

**Authors:** Le Manh Cuong, Ha Van Quyet, Tran Manh Hung, Nguyen Ngoc Anh, Tran Thu Ha, Vu Van Du, Do Van Loi, Ha Huu Hoang Khai, Vu Duy Kien

**Affiliations:** 1 National Hospital of Traditional Medicine, No. 29 Nguyen Binh Khiem Street, Hanoi, Vietnam; 2 Hanoi Medical University, No. 1 Ton That Tung Street, Hanoi, Vietnam; 3 Bach Mai Hospital, No. 78 Giai Phong Street, Hanoi, Vietnam; 4 National Hospital of Obstetrics and Gynecology, No. 43 Trang Thi Street, Hanoi, Vietnam; 5 OnCare Medical Technology Company Limited, No. 77/508 Lang Street, Hanoi, Vietnam

**Keywords:** High-resolution anorectal manometry, Anorectal manometry, Normal values, Healthy young people, Vietnam

## Abstract

**Background:**

High-resolution anorectal manometry (HRAM) has been developed to improve measurement of anorectal functions. This study aims to identify normal HRAM values in healthy young Vietnamese adults.

**Methods:**

We conducted a cross-sectional study at the National Hospital of Traditional Medicine (Hanoi, Vietnam) from July through December 2014. Healthy young adults were invited to participate in the study. All anorectal measurement values were performed using the ISOLAB high-resolution manometry system. Differences between groups were analyzed using Student’s t-tests.

**Results:**

Thirty healthy young adults, including 15 males and 15 females aged 19–26 years, were recruited. Mean functional anal canal length was 3.4 ± 0.5 cm (range: 2.4–4.8 mm). Mean maximum resting pressure, mean maximum squeezing pressure, mean maximum coughing pressure, and mean maximum strain pressure were 65.5, 168.0, 125.9, and 84.2 mm Hg, respectively. All anal pressure values were significantly different between males and females. For rectal sensation measurements, only the volume at first sensation was significantly higher in males than in females.

**Conclusions:**

This study provides normal HRAM value for healthy young adults in Vietnam. Sex may influence anal pressure and first rectal sensation values in this cohort. Further studies should be conducted in order to improve the quality of HRAM normal values and to confirm the effects of sex.

## Introduction

Anorectal manometry (ARM) is considered a leading method for describing anal and rectal functions [1, 2]. ARM measurements include functional anal canal length, anal pressure, defecation index, rectoanal inhibitory reflex (RAIR), and rectal sensation values, which, together, provide a description of comprehensive anorectal functioning [3–5]. Conventional ARM was widely used to diagnose and monitor anorectal disorders, although measurements varied in a manner that could affect diagnosis [6, 7]. As such, high-resolution anorectal manometry (HRAM) has been developed over the last ten years as an improved method for measuring anorectal function [2]. HRAM provides a comprehensive topographic and colorimetric mapping of the anorectal area. To accomplish this, the ARM sensor system and computer software were improved in order to record values of ARM over time and across locations by various companies whose HRAM technologies may include varying features, including differing numbers of pressure-sensitive elements, or various analytical software packages [2, 8, 9].

HRAM can aid in the detection of common anorectal disorders including fecal incontinence, constipation, and Hirschsprung’s disease [2]. However, HRAM values are suspected to fluctuate, affecting diagnosis in several anorectal diseases. Therefore, it is necessary to determine baseline HRAM values ​​in healthy people to be used in comparison with values from anorectal patients. Previous studies from various countries have reported related values [8, 10–12]. In Vietnam, HRAM technique is relatively common, and a study comparing HRAM values in patients before and after doppler-guided transanal hemorrhoidal dearterialization has previously been published [13]. However, there are no known studies regarding HRAM values ​​in healthy people. Moreover, there are no known studies investigating HRAM values ​​specifically in healthy young adults. As such, the aim of this study is to identify normal HRAM values in healthy young Vietnamese adults.

## Methods

We conducted a cross-sectional study at the National Hospital of Traditional Medicine (Hanoi, Vietnam). The study was approved by the ethics committee of the Military Medical University, Vietnam by the Decision no. 2858/QD-HVQY dated 23/11/2010. The study was conducted in accordance with relevant guidelines and regulations. Informed consent was obtained from all participants. This study was implemented from July through December 2014 as part of a larger research project investigating the use of doppler-guided transanal hemorrhoidal dearterialization (THD) in treating patients with hemorrhoid disease grades III and IV. A previous study was conducted that reported on anorectal HRAM values among hemorrhoidal patients before and after THD [13]. In the current study, all participants were healthy volunteers recruited from the pool of medical students at Vietnam University of Traditional Medicine in Hanoi. We provided study participants with all information regarding study aims, objectives, and possible anorectal HRAM adverse effects. All participants provided written consent before participating in the study.

We calculated the study sample size according to the formula for estimating a population mean proposed by the World Health Organization [14]. We assumed a maximum resting pressure of 69.1 mm Hg, according to the results of a previous study [11]. In addition, we assumed a population standard deviation of the maximum resting pressure of 12.5 mm Hg. With a significance level of 0.05 and a relative precision of 10 %, we calculated that the minimum sample size was 13 people. Since we intended to compare anorectal HRAM values between males and females, we doubled the sample size and included a 15% non-response rate, suggesting a selection of 30 healthy volunteers, including 15 males and 15 females. Inclusion criteria for participants included normal defecation for at least three months prior to enrollment and normal physical abdominal and digital anorectal examinations. We excluded participants who had constipation, fecal incontinence, obstructed defecation, hematochezia, hemorrhoid, rectal mucus discharge, or any history of anorectal disease. We conducted digital rectal examinations for all participants to ensure that participants were free from any anorectal disorders or diseases.

We used an ISOLAB high-resolution manometry system (Standard Instruments GmbH, Karlsruhe, Germany) with a 6 mm diameter catheter probe and eight-channel sensors to measure anorectal HRAM values. We recorded HRAM values based on pressure and an electromyographical signal from the digestive tract. To extract anorectal HRAM values after measurement, we used ViMeDat software (Visible Medical Data; Standard Instruments GmbH, Karlsruhe, Germany), which integrates with the ISOLAB system. All participants underwent an enema (Fleet enema, C.B. Fleet Company, Inc., Lynchburg, VA, USA) approximately one hour before measurement. We measured maximum anorectal HRAM pressure values of participants during periods of resting, squeezing, pushing/straining, and coughing, and defined a defecation index as the ratio of the maximum rectal strain pressure to the minimum anal strain pressure. We requested the participant relaxed by lying without speaking for about 5 min to measure the pressure in the resting period. To measure the pressure in the squeezing period, the participant was asked to squeeze the anal canal tightly for 20 s. Then, the participant was instructed to bear down (as if to defecate) for 10–20 s to measure the pressure of the pushing period. Wes asked the participant to cough five to seven times to measure the pressure in the coughing period. In this study, the RAIR was assessed if anal relaxation was greater than 25%. Rectal sensation was measured using a rectal balloon filled with air in 10 mL increments up to a total of 300 mL, and was recorded at three volumes corresponding to participant reporting of first sensation, the desire to defecate, and the urge to defecate. All measurements of the HRAM values in this study were performed by the guidelines proposed by Rao et al. and Lee et al. [2, 15].

Analytical results of quantitative variables were presented as means, standard deviation, and range (minimum and maximum). Differences between males and females were analyzed using Student’s t-test. We conducted all data analyses using STATA version 14.0 (Stata Corp, College Station, TX, USA). Statistical differences were considered significant at *p* values < 0.05.

## Results

A total of 30 healthy young adults were recruited to participate in the study, including 15 males and 15 females. Participants ranged from 19 to 26 years old, and 27/30 (90 %) of participants were aged 23 or 24 years old. Table 1 shows study participant HRAM values. Mean functional anal canal length was 3.4 ± 0.5 cm (range: 2.4–4.8 mm). Anal pressure values included mean maximum squeezing pressure at 168.0 ± 61.0 mm Hg and mean maximum resting pressure at 65.5 ± 13.9 mm Hg. Mean defecation index was 2.2 ± 0.9 (range: 1.3–4.8). Mean threshold volume to elicit RAIR was 17.0 ± 5.3 mL. Values for rectal sensation measurements were 19.7 mL, 46.7 mL, and 221.0 mL at the first sensation, desire to defecate, and urge to defecate, respectively.

**Table 1:** Anorectal HRAM parameters in 30 healthy study participants

	Mean (SD)	Min-Max
Functional anal canal length (cm)	3.4 (0.5)	2.4–4.8
*Anal pressure*		
Maximum resting pressure (mm Hg)	65.5 (13.9)	40.2–92.8
Maximum squeezing pressure (mm Hg)	168.0 (61.0)	78.3-352.4
Maximum coughing pressure (mm Hg)	125.9 (41.7)	42.1-228.4
Maximum strain pressure (mm Hg)	84.2 (36.3)	38.6-168.6
*Defecation index*	2.2 (0.9)	1.3–4.8
Threshold volume to elicit RAIR (mL)	17.0 (5.3)	10–30
*Rectal sensation*		
First sensation (mL)	19.7 (7.6)	10–40
Desire to defecate (mL)	46.7 (13.0)	30–90

*SD* Standard deviation, *RAIR* rectoanal inhibitory reflex

Table 2 presents HRAM values according to sex. The functional anal canal was significantly longer in males than in females (*p* < 0.01). In addition, all anal pressure values, including mean maximum anal squeeze pressure, mean maximum anal cough pressure, mean maximum anal strain pressure, mean maximum rectal cough pressure, and mean maximum rectal strain pressure, were also significantly higher in males than in females (all *p *values < 0.05). There were no significant differences in defecation index or threshold volume to elicit RAIR between males and females. For rectal sensation values, only the volume at first sensation was significantly higher in males than in females.

**Table 2:** Anorectal HRAM parameters in 30 healthy participants according to participant sex

	Male (n = 15)		Female (n = 15)		
	Mean (SD)	Min–Max	Mean (SD)	Min–Max	*p *value
Functional anal canal length (cm)	3.6 (0.5)	3.2–4.8	3.1 (0.5)	2.4–4.0	< 0.01
*Anal pressure*					
Maximum resting pressure (mm Hg)	73.4 (11.0)	52.3–92.8	57.6 (11.9)	40.2–78.8	< 0.01
Maximum squeezing pressure (mm Hg)	193.8 (65.5)	120.8–352.4	142.2 (44.6)	78.3–245.3	< 0.01
Maximum coughing pressure (mm Hg)	139.4 (48.8)	42.1–228.4	112.3 (28.6)	66.1–175.2	0.03
Maximum strain pressure (mm Hg)	95.6 (36.9)	44.2–168.0	72.7 (32.9)	38.6–168.6	0.04
Defecation index	2.0 (0.7)	1.3–3.7	2.3 (1.2)	1.3–4.8	0.78
Threshold volume to elicit RAIR (mL)	18.0 (5.6)	10–30	16.0 (5.1)	10–20	0.16
*Rectal sensation*					
First sensation (mL)	22.7 (7.0)	10–40	16.7 (7.2)	10–30	0.01
Desire to defecate (mL)	49.3 (14.4)	30–90	44.0 (11.2)	30–70	0.13
Urge to defecate (mL)	225.3 (66.1)	120–350	216.7 (64.8)	120–360	0.36

*SD* Standard deviation, *RAIR* rectoanal inhibitory reflex

## Discussion

This is the first study in Vietnam to explore typical HRAM values among healthy young adults and thus sets standard baseline values against which to compare in future studies. We show that males tended to have higher HRAM values than did females. Furthermore, we suggest that the availability of normal HRAM values will improve future diagnosis and treatment for patients with anorectal diseases.

Functional anal canal length was one indicator measured and allows comparison to various previous studies. Measured functional anal canal length was similar in our study to results reported by Carrington et al. [8], but much higher than those reported by Jorge et al. [16]. These differences may relate to measurement protocol or to sample size. Furthermore, functional anal canal length values as measured by HRAM may be affected by rectal pressure. When rectal pressure is low, functional anal canal length measurements may be inaccurate due to external artifacts. Vollebregt et al. demonstrated that a rectal pressure of 20 mm Hg or higher is optimal for measuring functional anal canal length [17]. As there is no current specific guideline for controlling rectal pressure during functional anal canal length measurement, it is possible that results from various studies cannot be meaningfully compared. However, all previous studies indicated significant differences in functional anal canal length between men and women; specifically, it was often higher in men than that in women [8, 16, 17]. Although functional anal canal length measurement may not be expected to have much diagnostic value in patients with fecal incontinence or constipation, it may still be useful for detecting anomalies in those patients. We suggest that studies that include a larger sample size should be conducted in order to learn more about functional anal canal length association with various anorectal disorders or diseases.

In this study, we measured baseline anorectal pressure values in healthy young adults, something which reflects the function of the anorectal muscles, including maximum resting pressure, maximum squeezing pressure, maximum coughing pressure, and maximum strain pressure values. Although previous studies have often reported these parameters, previously-reported values may not be as complete as those in our study, or may include other pressure values [8–12, 18, 19]. Although absolute anal pressure values in our study differ from those reported in previous studies, our results maintain previously-reported patterns. Specifically, mean maximum squeezing pressure was the highest pressure value, while mean maximum resting pressure was the lowest. We also found wide variability between minimum and maximum values for each parameter [8–12, 18, 19]. Whereas our study participants were of a similar age, and age did not affect our study results, we did find a statistically significant difference in anal pressure parameters between male and female study participants. This result is consistent with previous studies indicating that mean anal pressure values tended to be higher in males than in females [2, 8, 10, 19, 20]. As anal pressure is induced by the internal anal sphincter, external anal sphincter, and soft tissue of the anal canal [21–23], anatomical and physiological differences between males and females likely induce significant differences in measured anal pressure values [24]. Although there was wide variability and overlap between sexes of anal pressure measurements, understanding sex-related differences of anal pressure parameters may help improve anorectal disorder diagnosis and research. As there are currently no guidelines regarding the limits of these indicators, using standard protocols [2, 15, 25] and measuring values in healthy people may improve interpretation of study results and diagnosis in specific anorectal medical disorders [8, 12, 20, 26, 27].

The defecation index is defined as the ratio between the intrarectal pressure and anal sphincter residual pressure. Rectal pressure should thus generally exceed anal pressure in healthy adults [2]. In this study, we found that all defecation index values were greater than 1.3. In addition, we found no statistically significant difference in defecation index between males and females. Both of these results were consistent with previous studies [10, 19], although the defecation index was less commonly reported in studies of healthy people, regardless of its potential to indicate functional defecation disorder [28, 29]. Several studies indicated that some healthy people displayed dyssynergic defecation during HRAM measurement [28, 29].

RAIR indicates the anal reflex response and as such has been used to rule out a number of diseases, including megacolon and Hirschsprung’s disease [30, 31]. In our study, RAIR occurred in all study participants, indicating that all participants had the anal reflex response, although the threshold to elicit RAIR varied among participants. We found that the mean volume to elicit RAIR was 17 mL and was not significantly different between males and females. This result is similar to that of Coss-Adam et al., although those authors used high-definition anorectal manometry (HDAM-3D) [19]. Although no previous studies used HRAM to report on the threshold to elicit RAIR [8, 12, 20, 26, 27], we believe that it is an important parameter to aid research and disease diagnosis.

With regards to rectal sensation, our results differed from those of previous studies [12, 20]. Noelting et al. did not report any difference in rectal sensation between males and females [12], whereas Kritasampan et al. reported that the threshold volume to elicit the urge to defecate was significantly higher in males than in females [20]. Using HDAM-3D, Li et al. reported no significant differences between males and females [11], although Coss-Adam showed significant differences between males and females at the threshold volumes of desire to defecate and urgency to defecate [19]. These differences in reporting could be due to measurement protocols or the use of different measuring equipment. In addition, previous studies had a small sample size, so results may diverge from, and not reflect, actual population values. Studies with a larger sample size may thus be needed to improving knowledge about factors related to rectal sensation.

We are aware of some limitations of this study. The sample size was relatively small, something that may affect statistical interpretation. In addition, this was a single-center study, and our results may not be generalizable to the entire population. Beyond this, several other HRAM measurements that are important in assessing anorectal functions were not included in this study, and, although we realize that there are many other factors associated with anorectal function, we did not include those factors here. We conducted this study before the time of London classification for disorders of anorectal function published [32], so we could not apply some recommendations suggested by this protocol, which might limit comparison with other studies. Moreover, this is a cross-sectional study, so interpreting results to indicate causal relationships may not be recommended.

## Conclusions

This study establishes normal values of HRAM in young healthy adults in Vietnam. Sex influenced HRAM parameters, including functional anal canal length and the threshold volume of first sensation. We recommend implementing a study with a larger sample size to further confirm HRAM values in the Vietnamese population. In addition, a multicenter study may be needed in order to develop a standard set of normal HRAM values which could indicate baseline values for comparisons useful in the diagnosis and treatment for patients with anorectal medical disorders.

## Data Availability

The datasets generated during and/or analyzed during the current study are available in the Figshare repository: 10.6084/m9.figshare.12991217.v1.
